# Pomegranate seeds: a comprehensive review of traditional uses, chemical composition, and pharmacological properties

**DOI:** 10.3389/fphar.2024.1401826

**Published:** 2024-07-11

**Authors:** Jian Wang, Mengjie Sun, Jian Yu, Jinglong Wang, Qinghua Cui

**Affiliations:** ^1^ College of Food Science and Pharmaceutical Engineering, Zaozhuang University, Zaozhuang, China; ^2^ College of Pharmacy, Shandong University of Traditional Chinese Medicine, Jinan, China

**Keywords:** pomegranate seeds, chemical composition, unsaturated fatty acids, pharmacological effects, anti-tumor activity

## Abstract

Pomegranate seeds (PS) are the dried seeds derived from pomegranate fruit, accounting for approximately 20% of the fruit’s total weight, and are a by-product of pomegranate juice extraction. These seeds hold significance in traditional medicine among Uyghurs and Tibetan cultures, featuring diverse clinical applications within traditional Chinese medicine. These applications include management of gastric coldness and acidity, abdominal distension, liver and gallbladder fever, and pediatric enteritis. PS demonstrates properties such as stomach tonicity, qi regulation, analgesia, and anti-inflammatory effects. Extensive research underscores the richness of PS in various phytochemical compounds and metabolites, notably unsaturated fatty acids (particularly linolenic acid and linoleic acid), phenolic compounds tocopherols, proteins, and volatile oils. Notably, among these bioactive compounds, punicic acid (PA), found within PS, demonstrates potential in the prevention and treatment of cancers, diabetes, obesity, and other ailments. Despite extensive literature on pomegranate as a botanical entity, a comprehensive review focusing specifically on the chemical composition and pharmacological effects of PS remains elusive. Therefore, this review aimed to consolidate knowledge regarding the medicinal properties of PS, summarizing its chemical composition, traditional uses, and pharmacological effects in treating various diseases, thereby laying a foundation for the advancement and application of PS in the field of pharmacology.

## 1 Introduction

Pomegranate (*Punica granatum* L.), a member of the family Punicaceae, is a deciduous shrub or small tree with a longstanding history of cultivation in China. The first documented medicinal use of various pomegranate parts, including the peel, seeds, flowers, leaves, and roots, dates back to the Han Dynasty, as recorded in Min-Yi-Bie-Lu (名医别录Han Dynasty). Therefore, pomegranate has a very long history of use not only in traditional Chinese medicine but also in diverse clinical practices among Tibetans, Uyghurs, Miaos, and other ethnic groups. Apart from its nutritional significance, pomegranate has important medicinal attributes. Research indicates that different components of the pomegranate, such as the juice, pericarp, seeds, flowers, leaves, and peels, are rich in biologically active compounds with anti-diabetic, anti-tumor, anti-inflammatory, anti-malarial, and anti-fibrotic properties, which are widely recognized and utilized globally (Maphetu et al., 2022; Yisimayili and Chao, 2022). Moreover, studies have revealed that the bark of the pomegranate tree is a natural green corrosion inhibitor (Marsoul et al., 2020). Pomegranate also finds clinical application in Hmong medicine for treating conditions such as chronic diarrhea, dysentery, and roundworms. Furthermore, pomegranate flowers are used to treat nosebleeds, whereas whole pomegranate dried fruit is used in Mongolian medicine for treating conditions such as gastritis, indigestion, and abdominal distension. The Pharmacopoeia of the People’s Republic of China (2020 edition) acknowledges the efficacy of pomegranate peel in astringent and antidiarrheal, hemostatic, and anthelmintic capacities. Various pharmaceutical properties attributed to pomegranate peels include anti-proliferative, anti-inflammatory, and anti-cancer effects (Wong et al., 2021). Pomegranate juice, which is rich in antioxidants, demonstrates cholesterol oxidation resistance, atherosclerosis prevention, anti-inflammatory and anti-aging effects, along with potential to prevent Alzheimer’s disease and diabetes (Sreeja et al., 2012). Consequently, the utilization of pomegranate by-products continues to expand, enhancing the economic efficiency of the pomegranate industry.

Pomegranate seeds (PS), derived from the pomegranate fruit in dried form, constitute approximately 20% of its total fruit weight (Gao et al., 2023), serving as the primary by-product of pomegranate juice processing. Despite this, PS are relatively under-researched and underutilized, leading to substantial amounts of annual waste estimated at 70,000–100,000 tons. Although, PS represent a significant amount among waste products in the fruit processing industry, they hold considerable value as a resource for pharmaceuticals and nutraceuticals (Khoddami et al., 2014). In Tibetan medicine, PS is commonly known as“Saizhu”and is recognized for its sweet and sour taste, warm properties, and therapeutic effects in ailments of stomach, digestion, and lungs. With heat-clearing and damp-drying effects, PS are used in traditional medicine for treating conditions such as hemorrhage, dampness-heat syndrome, and other diseases (Huo et al., 2023). Research indicates diverse pharmacological effects associated with PS, including anti-tumor and anti-osteoporosis properties, possibly attributable to its constituents such as unsaturated fatty acids, phenols, sterols, proteins, and volatile oils (Min et al., 2010; Kaseke et al., 2021; Guzmán-Lorite et al., 2022a). Pomegranate seed oil (PSO), extracted from PS, constitutes 12%–20% of PS’s total weight and is notably rich in punicic acid (PA), a rare isomer of conjugated linoleic acid known for its efficacy against breast cancer, prostate cancer, diabetes, and obesity (Aruna et al., 2016; Zielińska et al., 2022). PA exhibits promising potential as an alternative treatment (Franczyk-Żarów et al., 2023), and the extraction of PSO not only minimizes waste but also enhances the economic and health benefits associated with pomegranate (Fourati et al., 2020).

The full potential of PS remains largely untapped due to an inadequate understanding of its value and a dearth of development in its applications. Despite being a valuable resource, PS have not been sufficiently exploited for their benefits. Although some recent reviews of pomegranate research have briefly addressed studies related to PS, a comprehensive examination of their chemical composition is yet to be achieved (Kori et al., 2020; Ge et al., 2021; Maphetu et al., 2022). In recent years, several studies have investigated the chemical composition of PS (Hernández-Corroto et al., 2022; Iriti et al., 2023); however, these studies have focused on only one or a few components of PS. This paper presents a comprehensive and systematic review of the chemical composition of PS. Moreover, there is a notable absence of systematic summaries concerning the pharmacological effects of PS, as well as a lack of comprehensive reviews detailing their traditional uses, clinical applications, and chemical metabolites. The absence of such reviews hampers a broader and updated perspective. Hence, there is a need for systematic and comprehensive reviews that could provide a foundation for leveraging PS effectively and to stimulate further research and development of PS applications in the pharmaceutical, health product, food, and other industries.

## 2 Methods

Literature search was conducted using the keywords “pomegranate seed,” “pharmacological properties, or activities, or effects, or roles,” “ethnopharmacology,” “traditional uses,” “botanical characteristics,” “chemical composition” in major scientific literature databases such as PubMed, Web of Science, Wiley, Francis & Taylor, Hindawi, SciFinder, Science Direct, Springer, ACS, CNKI, Google Scholar, and Baidu Scholar. Additionally, books, MSc theses, and Ph.D. From approximately 200 identified studies, a total of 115 studies, which met the inclusion criteria, were preserved in this survey.

## 3 Botanical characteristics

Pomegranate (*P. granatum* L.), a member of the Punicaceae family, thrives in tropical and subtropical regions worldwide. Originating from ancient Mediterranean areas characterized by cool winters and warm dry summers, this climate fosters optimal growth conditions for the plant (Sarkhosh et al., 2020). Cultivated across diverse regions, the pomegranate assumes various names. In Asian countries such as China, Georgia, and Afghanistan, a wide array of pomegranate varieties has been reported (Ge et al., 2021).

The pomegranate plant reach heights of 4–5 m, adorned with thorny branches (Sarkhosh et al., 2020). Additionally, the plant features flaky bark and glossy, crinkled petal leaves (Guerrero-Solano et al., 2020). Upon ripening, the fruit forms a berry measuring approximately 5–12 cm in diameter, boasting a round shape and thick, reddish skin. Each fruit contains anywhere from 200 to 1400 seeds enveloped in water-laden pulp (Stover and Mercure, 2007), which varies in color from white to deep red or purple.

## 4 Traditional uses

Bai-Di-Yi-Yao-Shu (拜地依药书) documented pomegranate peel, seeds, and flowers as possessing a sour and astringent taste, prescribed for managing diarrhea, dysentery, and facilitating wound healing (Editorial Board of Chinese Materia Medica of National Administration of Traditional Chinese Medicine, 2005). Jing Zhu Ben Cao (晶珠本草) indicates that PS can effectively address stomach ailments, warm the stomach, and alleviate symptoms of Peigen disease. In Si Bu Yi Dian (四部医典), pomegranate seeds are hailed as “the king of warm medicine,” utilized for fortifying the stomach, enhancing appetite, and managing “Peigen” cold disease.

In traditional Uyghur and Tibetan medicine, PS serves primarily in the treatment of gastric disorders and is esteemed for its bioactive metabolites and functional lipid abundance. Contemporary research demonstrates that pomegranate seeds exhibit antioxidant, anti-inflammatory, anti-tumor, hypoglycemic, hypolipidemic, and cardiovascular protective properties (Chen et al., 2023a). Traditional medicinal applications of PS are summarized in Table 1.

**TABLE 1:** Traditional medicinal uses of pomegranate seeds.

Book title	Clinical use
Zang-Yao-Pei-Fang-Xin-Bian	Warming the stomach, treatment of dyspepsia and loss of appetite; treatment of “Hanlong” and “Feilong” disease; blood enrichment
Jing-Zhu-Ben-Cao	Treatment of stomach disease, warming the stomach, and treatment of “Peigen” disease
Si-Bu-Yi-Dian	Strengthening the stomach, stimulating the appetite, and treating “Peigen” cold disease
Xi-Zhang-Chuan-Tong-Yi-Xue-Gai-Shu	Strengthening the spleen and treatment of stomach cold, dyspepsia, and abdominal distension
Chang-Yong-Zhong-Yao-Zhi	Treatment of “Peigen” disease and stomach diseases, such as stomach cold
Zhong-Guo-Zang-Yao	Treatment of gastrectasis, severe pain caused by liver disease, and “Peigen” disease
Zang-Yi-Yao-Xuan-Bian	Treatment of diseases of the large intestine (cold syndrome) and kidneys
Zang-Yao-Biao-Zhun	Strengthening the stomach, treatment of loss of appetite, stomach cold pain, tumescence, and dyspepsia
Bai-Di-Yi-Yao-Shu	Treatment of diarrhea and dysentery, and promotion of wound healing

As per Zhong Hua Ben Cao’s Tibetan Medicine Volume (中华本草·藏药卷), Shunqi thirteen-flavored San, incorporating pomegranate, dried ginger, pepper, *Piper longum*, nutmeg, round cardamom, Amomum tsao-ko, safflower, *Cinnamomum cassia*, myrobalan, Halite, Halite Violaceous, and *Nigella glandulifera*, is prescribed for intestinal distension and bloating (State Administration of Traditional Chinese Medicine, 2002). Pomegranate Stomach Pill composed of PS, Cinnamomum cassia, Piper longum, safflower, and round cardamom; proven effective in treating gastroenteritis (Kong et al., 2021). In contemporary medicine, PS is often combined with other Chinese medicines to enhance their efficacy in stomach and kidney warming and in managing cholecystitis and enteritis (Supplementary Table S1). Top of Form.

## 5 Chemical composition

PS contains a wide array of phytochemicals and bioactive compounds. Li et al. (2020) effectively identified flavonoids and phenolic acids within PS. Furthermore, in 2023, the same research team identified fatty acids, sterols, and other metabolites in PS (Li et al., 2023). The principal metabolites present in PS include fatty acids, flavonoids, phenolic acids, proteins (Hernández-Corroto et al., 2022), volatile oils (Min et al., 2010), phytosterols, and others such as squalene, carotenoids, and vitamin E (Iriti et al., 2023).

### 5.1 Fatty acids

**FIGURE 1:**
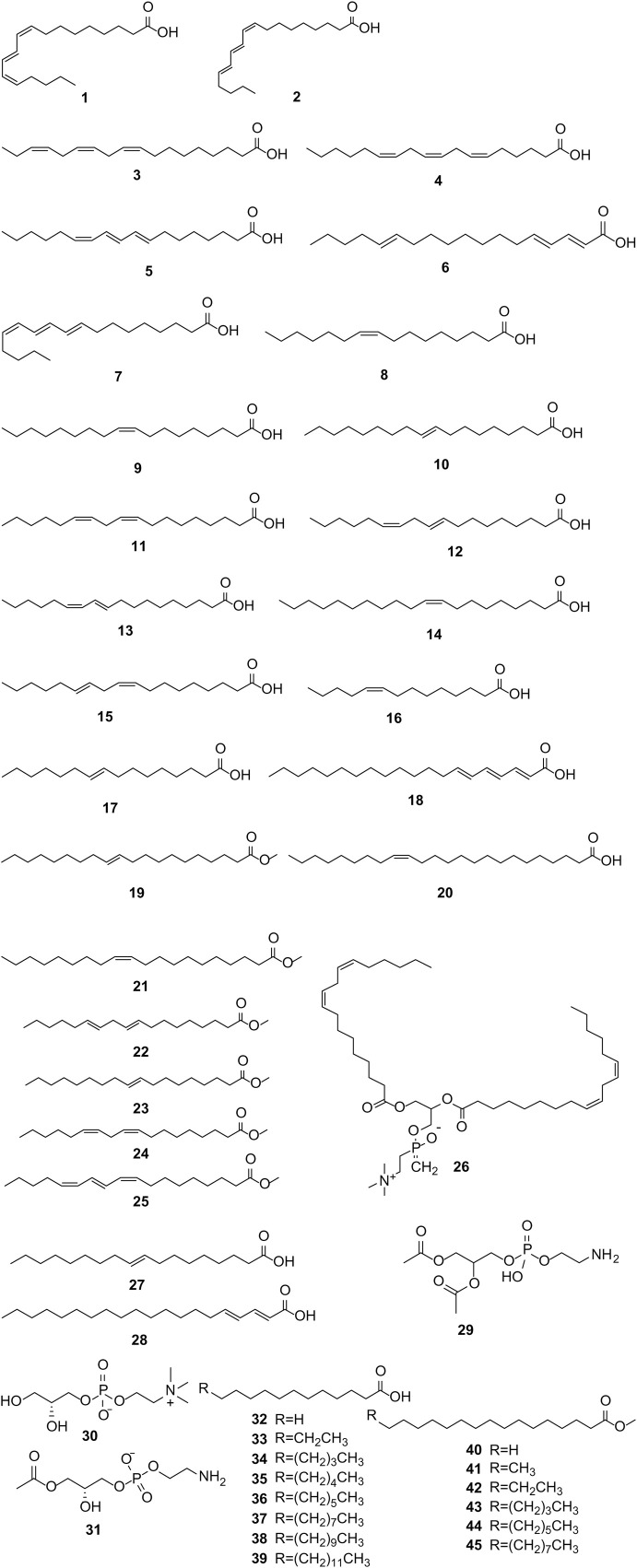
(Continued).

PS is abundant in various fatty acids (Eikani et al., 2012), characterized by significant levels of monounsaturated fatty acids (MUFAs) and polyunsaturated fatty acids (PUFAs), and lesser quantities of saturated fatty acids (SFAs), as elucidated by Siano et al. (2016). The total lipid content in PS ranges from 7.9% to 16%, with PSO particularly rich in conjugated linolenic acids (CLnAs), notably PA, constituting 74%–85% of the total fatty acid content. Other prominent fatty acids include oleic, linoleic, and palmitic acids, alongside certain phospholipids (Verardo et al., 2014). The fatty acid composition of PS is detailed in Supplementary Table S2, with the structural formulas of these fatty acids depicted in Figure 1.

### 5.2 Phenolic metabolites

**FIGURE 2:**
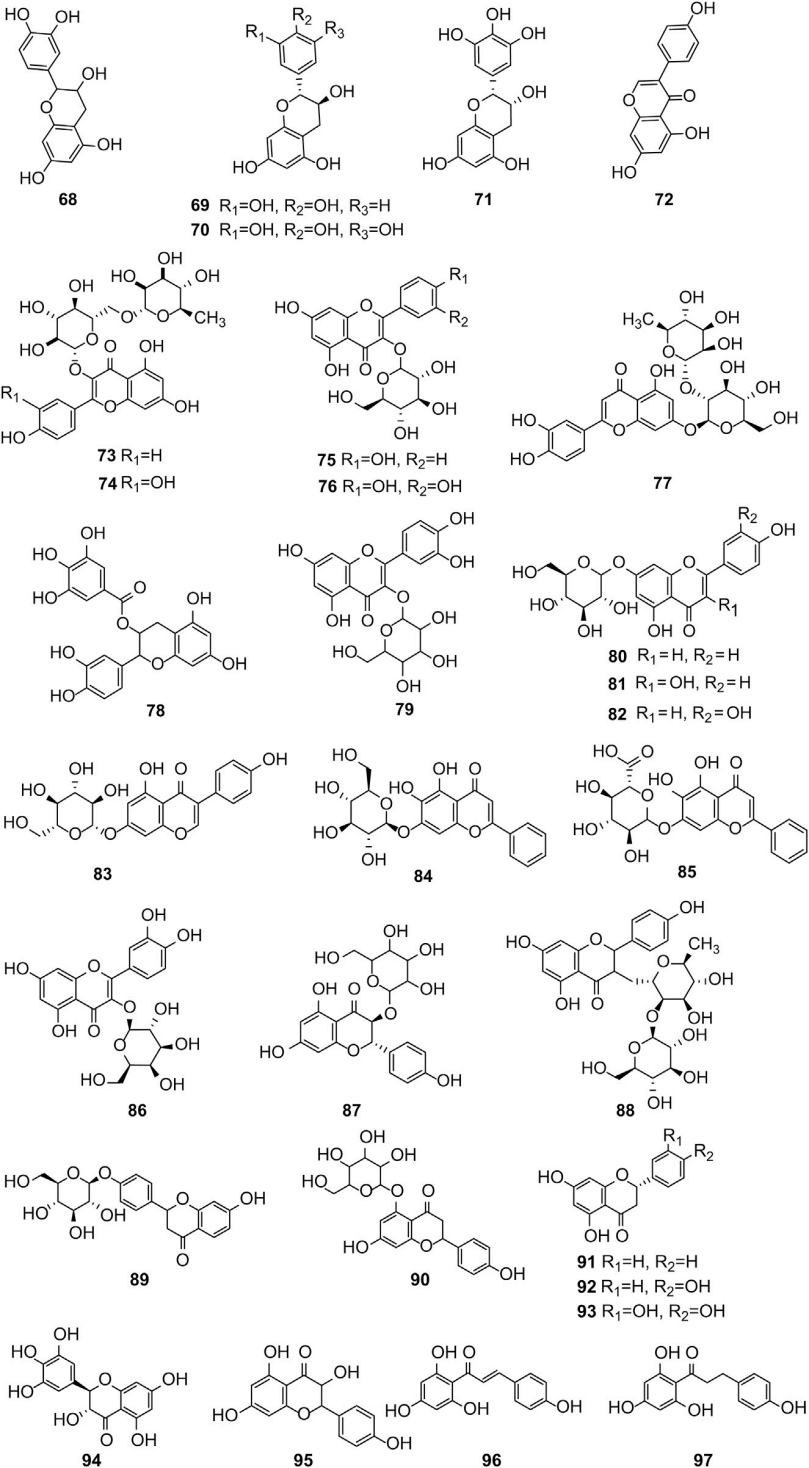
Structures of the flavonoids in pomegranate seeds.

**FIGURE 3:**
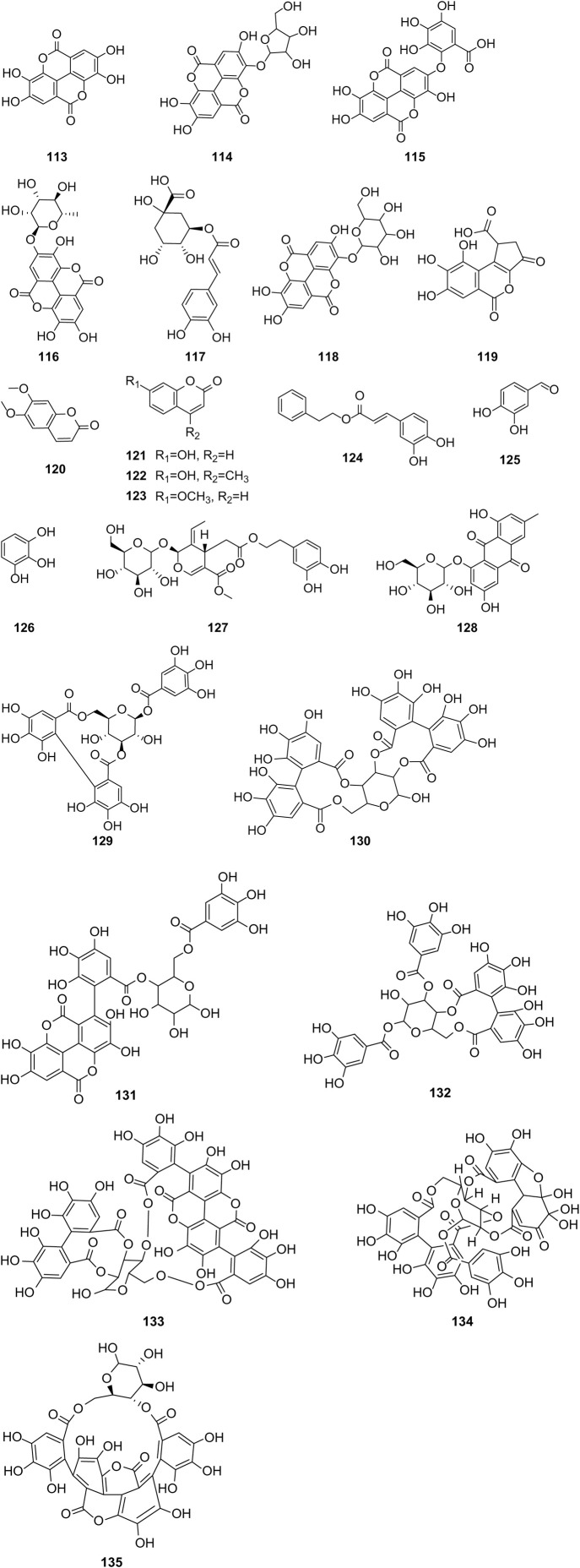
(Continued).

Hernández-Corroto et al. (2022) identified 30 different phenolic metabolites. Among these, ellagic acid emerges as a key phenolic metabolite in PS, renowned for its anti-cancer, anti-inflammatory, antioxidant, and hypolipidemic pharmacological effects (Ranjha et al., 2021). Ambigaipalan et al. (2017) identified 47 phenolic metabolites in PS, encompassing various flavonoids renowned for their antioxidant activity. Notably, naringenin hexoside and catechins were the most abundant flavonoids. The flavonoid composition of PS is detailed in Supplementary Table S3, with structural formulas presented in Table 2 and Figure 2. Phenolics in PS, excluding flavonoids, are shown in Supplementary Table S4, while the structural formulas of phenolic metabolites other than flavonoids are presented in Tables 3, 4 and Figure 3.

**TABLE 2:** Structures of the flavonoids in pomegranate seeds.

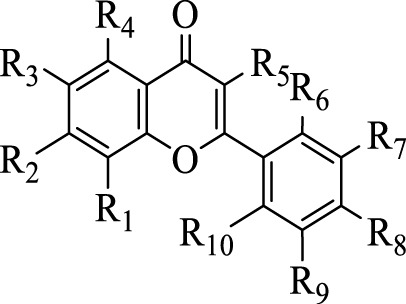										
Compd	R_1_	R_2_	R_3_	R_4_	R_5_	R_6_	R_7_	R_8_	R_9_	R_10_
46	H	H	H	H	H	H	OCH_3_	H	H	OCH_3_
47	H	OCH_3_	H	H	H	OCH_3_	OCH_3_	H	H	H
48	H	H	OCH_3_	H	OCH_3_	H	OCH_3_	OCH_3_	H	OCH_3_
49	H	H	OCH_3_	H	OCH_3_	OCH_3_	OCH_3_	H	H	H
50	H	H	H	H	H	OCH_3_	OCH_3_	H	H	H
51	H	OCH_3_	OCH_3_	H	H	H	OCH_3_	OCH_3_	H	H
52	H	OCH_3_	H	OCH_3_	OCH_3_	H	OCH_3_	OCH_3_	H	H
53	H	H	H	H	OCH_3_	H	OCH_3_	OCH_3_	H	OCH_3_
54	OCH_3_	OCH_3_	OCH_3_	OH	H	H	OCH_3_	OCH_3_	OCH_3_	H
55	H	OCH_3_	H	H	H	H	OCH_3_	OCH_3_	OCH_3_	H
56	OCH_3_	OCH_3_	H	H	OCH_3_	H	H	H	H	OCH_3_
57	H	H	OCH_3_	H	OCH_3_	H	OCH_3_	OCH_3_	H	H
58	H	OCH_3_	H	OCH_3_	OCH_3_	H	H	H	H	H
59	OCH_3_	OCH_3_	H	H	OH	H	H	H	H	OCH_3_
60	OCH_3_	OCH_3_	H	H	H	H	OCH_3_	OCH_3_	H	H
61	H	OH	H	OH	OH	H	H	OH	H	H
62	H	OH	H	OH	H	H	H	OH	H	H
63	H	OH	H	OH	OH	H	OH	OH	H	H
64	H	OH	H	OH	H	H	OH	OH	H	H
65	OCH_3_	OCH_3_	OCH_3_	OCH_3_	H	H	OCH_3_	OCH_3_	H	H
66	H	OH	OH	OH	H	H	H	OH	H	H
67	H	OH	H	OH	OH	H	OH	OH	OH	H

**TABLE 3:** Structures of the phenolic compounds other than flavonoids in pomegranate seeds (1).

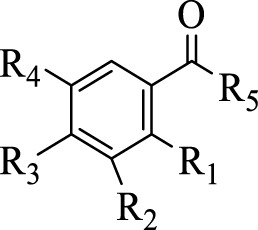					
Compd	R_1_	R_2_	R_3_	R_4_	R_5_
98	H	OH	OH	OH	OH
99	H	H	OH	OCH_3_	OH
100	H	OH	OH	OH	OCH_3_
101	H	OH	OH	OH	OCH_2_CH_3_
102	H	H	OH	OH	OH
103	OH	H	H	OH	OH
104	H	OH	OH	OH	O(CH)_2_CH_3_
105	OH	H	OCH_3_	H	CH_3_

**TABLE 4:** Structures of phenolic compounds other than flavonoids in pomegranate seeds (2).

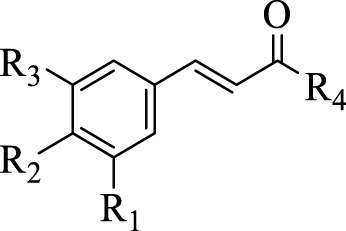				
Compd	R_1_	R_2_	R_3_	R_4_
106	H	H	H	OH
107	H	OH	H	OH
108	H	OH	OH	OH
109	H	OH	OCH_3_	OH
110	OH	OCH_3_	H	OH
111	H	OH	OCH_3_	OCH_3_
112	OCH_3_	OH	OCH_3_	OH

### 5.3 Volatile oil metabolites

Min et al. (2010) employed GC/MS to examine the volatile oil metabolites in sour and sweet PS, identifying 14 and 15 fractions, respectively. Both varieties of PS contained volatile oils such as geranial, carvacrol, and eucalyptol, albeit with notable differences in relative contents between them.

### 5.4 Other classes

PS contains 10%–20% protein (Talekar et al., 2018). Elfalleh et al. (2012) investigated the free amino acid and protein contents of Tunisian and Chinese PS, revealing 18 common free amino acids in both varieties, including all essential, sulfur-containing, and aromatic amino acids, with glutamic acid, arginine, and aspartic acid prevailing as the major amino acids. These amino acids are often lacking in most foods (Bar-Ya’akov et al., 2019). Guzmán-Lorite et al. (2022b) devised an environmentally friendly method for the comprehensive recovery of proteins from PS using natural deep eutectic solvent choline chloride and glacial acetic acid (ChCl:HAC), alongside continuous pressurized liquid extraction under alkaline conditions, facilitating the eco-friendly and complete retrieval of proteins from PS.

Additionally, PS comprises steroids, carotenoids, tocopherols, trace elements, and other metabolites. Górnaś and Rudzińska (2016) identified numerous phytosterols in PSO, including glycosterol, stigmasterol, glutosterol, sterol, 7-stigmasterol, and cyclic oxysterol. Furthermore, PS is rich in trace elements. Using flame atomic absorption spectrometry, Yin et al. (2011) detected nine trace elements in sour and sweet PS, including Cu, Mn, Zn, Fe, Cr, K, Ca, Na, and Mg.

## 6 Pharmacological activities

Numerous studies have showcased the anti-tumor effects (Costantini et al., 2014) and anti-osteoporosis properties (Yogesh et al., 2020) of PS. Esther Lydia et al. (2020) developed a functional yogurt by employing PSO as a reducing agent to cap gold nanoparticles, demonstrating its *in vitro* antioxidant and anti-cancer activities.

### 6.1 Anti-tumor and anti-cancer effects

The primary metabolite in the hydrophilic extract of PSO is PA. *In vitro* experiments treating two breast cancer cell lines, MDA-MB-231 and MCF-7, with 0.5 μL and 0.6 μL of the extract led to an increase in the number of cells in the G0/G1 phase of the cell cycle compared to untreated cells. Moreover, treatment with higher doses of the extract showed more pronounced effects. Additionally, levels of vascular endothelial growth factor and pro-inflammatory cytokines decreased, indicating anti-inflammatory properties and preventive effects on tumor formation and metastasis (Costantini et al., 2014). Another *in vitro* study on human breast cancer cell lines MCF-7 and MDA-MB-468 using PSO yielded similar results (Mahmoudi et al., 2017). PSO inhibits the proliferative activity of breast cancer cells through various molecular proteins or signaling pathways, potentially by regulating the expression of Cox-2, Bcl-2, Bax, cystatinase-3 (zymosan), and P53 in cells (Fu et al., 2015).

Mete et al. (2019) conducted *in vitro* experiments to evaluate the toxic effects of PA on T98 cells. After 24 h of exposure to doses of 1, 5, 10, 50, and 100 μL/mL, they determined that the IC_50_ dose was 9.85 μL/mL. Applying the IC_50_ dose of PA to an *in vitro* wound model inhibited the migration and proliferation of T98 glioblastoma cells. Additionally, PA treatment reduced angiogenesis, tumor progression, and induced apoptosis. Notably, PA treatment significantly decreased the levels of PI3K and AKT1 in T98 cells, thereby inhibiting the proliferation of glioblastoma cells, possibly through the PI3K/Akt/mTOR signaling pathway.

Nasr et al. (2023) conducted *in vitro* experiments using fresh medium containing two-fold serial dilutions of PS and methanol extract of pomegranate peel, added to pre-cultured plates of human hepatocellular carcinoma cells (HepG2 cells) at a concentration of 0.1 mL/mL, respectively. Their findings revealed that pomegranate seeds exerted a significant toxic effect on HepG2 cells compared to peel extracts. Furthermore, there was notable cell cycle blockade and cell death in the G0/G1 and S phases, with cell cycle arrest and cell death not observed in the G2/M phase. Cell arrest was followed by an increase in reactive oxygen species (ROS) and MDA levels, alongside a decrease in superoxide dismutase (SOD), GSH, and catalase levels. Among apoptosis-related genes, the expression of pro-apoptotic genes (P53, Cy-C, Bax, casp-3) was significantly upregulated, while the expression of the anti-apoptotic gene (Bcl-2) was significantly downregulated.

Another study demonstrated that the total flavonoids in PS possess cancer-preventive effects by effectively eliminating nitrite, a precursor for nitrosamine synthesis, and inhibiting nitrosamine synthesis (Wang et al., 2018).

Astrocytic glioblastoma (GBM) is considered resistant to chemotherapy. The utilization of nanocarrier systems to traverse the blood-brain barrier has the potential to enhance therapeutic efficacy and mitigate the side effects of conventional chemotherapy. PSO nanoemulsions (n = 3) containing Laplacin were formulated via spontaneous emulsification solvent diffusion. These nanoemulsions were then utilized for the preparation of ketoprofen, a non-steroidal anti-inflammatory drug, followed by *in vitro* testing on C6 cells. The results of the study revealed that the nanoemulsions enhanced the photostability of the drug against UVC radiation and improved its solubility. Furthermore, these formulations have exhibited suitability for intravenous administration and have demonstrated significant activity against glioma cells *in vitro* (Ferreira et al., 2015).

### 6.2 Hypoglycemic effects

PA has been shown to exert anti-diabetic effects through various mechanisms, including the reduction of inflammatory cytokines, regulation of glucose homeostasis, and contains antioxidant properties (Khajebishak et al., 2019a). PSO exhibits good digestibility, bio-accessibility and anti-inflammatory properties (Bañares et al., 2023). Harzallah et al. (2016) conducted *in vitro* experiments on male C57Bl/6 mice, administering PSO (2 mL/kg/d) and assessing parameters such as body weight, body fat, energy expenditure, food and fluid intake, blood glucose, plasma insulin, and lipids. Their findings revealed that PSO improves insulin sensitivity. PSO’s therapeutic efficacy in patients with type 2 diabetes mellitus was investigated by Khajebishak et al. (2019b) in a randomized clinical trial involving 52 obese type 2 diabetes mellitus patients over an 8-week period. Participants were divided into an intervention group (n = 26; receiving three capsules containing 1 g of PSO per day) and a placebo group (n = 26; receiving equal amounts of paraffin). GLUT-4 gene expression and glycemic index were evaluated using standard methods, demonstrating an increase in GLUT-4 gene expression in diabetic patients without any observed side effects. However, further clinical studies are warranted to validate the findings. In a study by Wu et al. (2015b), the effect of PSO on plasma phospholipids in mice with type 2 diabetes mellitus was examined. They found that the pharmacological mechanism may involve the activation of AMP-activated protein kinase. Nevertheless, additional research is required to elucidate the underlying biological mechanisms.

### 6.3 Antioxidant, anti-inflammatory, and analgesic effects

PS serves as a rich source of various antioxidants, including flavonoids, PA, and α-tocopherol (Jing et al., 2012). Flavonoids found in PS neutralize free radicals and have strong DPPH scavenging activity. Notably, the concentration of DPPH significantly decreased with increasing levels of ethanol extract from PS (Al-Huqail et al., 2018). PSO also demonstrates antioxidant activity by scavenging free radicals and DPPH radicals in a dose-dependent manner (Harzallah et al., 2016). This antioxidant capacity is likely attributed to PA, a natural antioxidant known for its potent free radical scavenging activity (Rojo-Gutiérrez et al., 2021). PA has been shown to upregulate the expression of peroxisome proliferator-activated receptor (PPAR), thereby reducing oxidative damage and inflammation (Guerra-Vázquez et al., 2022). However, research by Đurđević et al. (2018) indicates that PSO exhibits strong antioxidant effects but is highly susceptible to oxidative deterioration when exposed to light, moisture, and oxygen. Nevertheless, the application of *Oliveria decumbens* essential oil (ODEO) has been shown to enhance the oxidative stability of PSO (Golmakani et al., 2021).

Li et al. (2018) conducted an experiment assessing the effects of PSO on aging model mice. They observed enhanced G6PD activity and increased NADPH content in the liver and kidney across all dose groups (75, 250, and 750 mg/kg). Administration of PSO reversed body weight loss, reduced MDA content in the liver, kidney, brain, and serum, increased GSH content, and improved the activities of T-AOC and the antioxidant enzymes SOD and GSH-Px. These findings suggest that PSO exerts significant antagonistic effects against D-galactose-induced oxidative stress in aging model mice, indicating its potential application in the development of anti-aging health foods. Hamouda and Felemban (2023) conducted an *in vivo* study administering PSO orally to rats at a dose of 250 mL/kg body weight daily for 21 days. Their results demonstrated that PSO reduced levels of collagenase, elastase, hyaluronidase, tyrosinase, cyclooxygenase-2, lipid peroxidation, and nitric oxide in the rats. Subsequently, they conducted a human study involving 60 women with skin problems, such as calluses on the hands, nail inflammation, and extra skin tags around the nails. Each participant received PSO for 21 days (250 mL/kg), confirming the results observed in the rat study. Moreover, incorporation of PSO nanocapsules into Laplacian films facilitated topical delivery of PSO and improved drug bioavailability in the treatment of atopic dermatitis (Ferrari Cervi et al., 2021). Ferreira et al. (2016) performed *in vivo* experiments with PSO-ketoprofen nanoemulsions on male adult Swiss mice. Mechanical anomalous pain testing using Von Frey Hair revealed that the duration of action of free ketoprofen was up to 6 h, whereas that of the nanoemulsions was up to 10 h, indicating prolonged anti-injury perception effects. Additionally, acute toxicity assessment based on alanine aminotransferase (ALT) and aspartate aminotransferase (AST) activity and urea levels after repeated use of the nanoemulsion over 7 days showed no toxic effects in the animals. The results suggest that the formulation holds promise as an alternative treatment for inflammatory and pain-related disorders such as arthritis.

Mizrahi et al. (2014) developed a nanodroplet formulation of PSO aimed at treating mice with genetic prion disease. Administering Nano-PSO via gavage five times (150 μL/day) to mice, they observed that Nano-PSO delayed the onset and progression of the disease within a shorter timeframe and at a lower dose compared to natural PSO. The treatment reduced lipid oxidation and neuronal loss, demonstrating neuroprotective effects. In another study, PS hydro-ethanolic extract (administered at doses of 200, 400, 800 mg/kg via gavage) was given to scopolamine-induced amnesic rats over a period of 3 weeks. During the third week, scopolamine was administered 30 min before conducting Morris water maze (MWM) and passive avoidance tests. The PS hydro-ethanolic extract notably reduced the time (up to 173%) and distance (up to 332%) required to reach the plateau during MWM learning (*p* < 0.001). Moreover, it led to a decrease in the expression of TNF-α, IL-1β, and AChE in hippocampal tissues (with maximum reductions of 114%, 137%, and 106%, respectively; *p* < 0.01). These findings indicated that PS could mitigate scopolamine-induced memory and learning deficits in rats by enhancing the function of the cholinergic system, suppressing oxidative stress, and modulating inflammatory factors (Akbarian et al., 2022).

### 6.4 Anti-osteoporotic effects

PSO extract is abundant in phytoestrogens and antioxidant metabolites. In a study by Sheng et al. (2016), PSO was administered daily via gavage to de-ovulated rats at doses of 0.95 g/kg and 1.27 g/kg. The results indicated a reduction in serum calcium and phosphorus levels, as well as alkaline phosphatase activity. Conversely, serum SOD levels increased, while malondialdehyde (MDA) levels decreased. Moreover, there was an increase in bone mineral density at various skeletal sites, including the mandible, mid-point of the femur, and distal end of the femur (Sheng et al., 2016). In another study utilizing bilateral de-ovulated female western albino rats as postmenopausal models, PSO n-hexane extract (administered at 500 μL/kg body weight/day) showed promising results in improving bone structure, density, and markers. This extract exhibited therapeutic effects in osteoporosis without adverse effects on lipid levels, uric acid levels, or liver function (Shaban et al., 2017). These findings suggest that PS could serve as a safe, effective, and cost-efficient alternative for preventing and treating osteoporosis in postmenopausal women. Furthermore, administration of 30 mg of pomegranate seed extract (PSE) (approximately 100–120 mg/kg) per day to de-ovulated rats led to significantly elevated levels of estradiol and notable improvements in vaginal atrophy and tibial thickness compared to those fed standard food. The results suggest that PS or its extracts may serve as effective complementary or alternative therapies for preserving bone mineral density and treating vaginal epithelial atrophy during menopause (Kaban et al., 2018).

Zhang et al. (2016) investigated the impact of aqueous extract of PS (AE-PS) on bone loss in glucocorticoid (GC)-induced osteoporosis (GIOP) mice. They administered AE-PS at a dose of 0.1 mg/kg/d for 12 weeks in combination with dexamethasone (DXM) and examined its effects on calcium homeostasis. The results revealed that AE-PS decreased bone loss, effectively curbed urinary calcium loss, and mitigated bone tissue loss in mice. In a subsequent study by Zhang et al. (2019), an *in vivo* experimental investigation was conducted using pure PSO at doses of 0.95, 1.27, and 1.59 g/kg via gavage in Sprague Dawley (SD) rats categorized into experimental groups 1, 2, and 3. Compared to group 1, groups 2 and 3 exhibited a reduction in calcium content and serum malondialdehyde (MDA) activity, along with an increase in SOD activity. These findings suggested that PSO protects cartilage by enhancing oxygen radical scavenging activity and suppressing oxidative stress. Moreover, it hindered cartilage degeneration in the knee joints of osteoarthritic rats and inhibited the deposition of calcium and phosphorus in the cartilage and subchondral bone, thereby promoting the restoration of chondrocyte elasticity. Liu et al. (2021) administered pure PSO in doses of 0.95 g/kg, 1.27 g/kg, and 1.59 g/kg to rats with osteoarthritis via gavage. They compared these doses with a model group that received 1.59 g/kg of normal saline via gavage. The mRNA expression of matrix metalloproteinase-1 in chondrocytes decreased significantly (*p* < 0.01), while mRNA expression of type II collagen (CoⅡ) increased significantly (*p* < 0.01). These results suggested that the mechanism of action of PSO in osteoarthritis may involve inhibiting cartilage matrix-degrading enzymes by down-regulating the mRNA expression of matrix metalloproteinase-1 in articular chondrocytes. This action slows down the breakdown of the articular cartilage matrix, promotes the recovery of cartilage elasticity, and improves cartilage degeneration by up-regulating the mRNA expression of type II collagen (Co II) in chondrocytes.

### 6.5 Anti-cardiovascular disease effects

Ambigaipalan et al. (2017) demonstrated that PS exhibits significant antioxidant activity, thereby safeguarding the cardiovascular system against free radical-induced damage. It inhibits copper-induced low-density lipoprotein-cholesterol peroxidation, reduces the activity levels of α-glucosidase and lipase, as well as mitigates blood pressure, cholesterol levels, and vascular inflammatory responses. These findings suggest that PS can effectively prevent cardiovascular and cerebrovascular diseases. Additionally, PS confers protective effects against methotrexate-induced alterations in serum oxidative stress (SOD and GPx) and lipids (total cholesterol, high-density lipoproteins, and low-density lipoproteins) in rats (Doostan et al., 2017). The consumption of PSO may enhance cardiovascular health by decreasing plasma total cholesterol and low-density lipoprotein (LDL) levels. Teh et al. (2019) conducted an *in vivo* experimental study by administering PSO to hamsters, demonstrating its efficacy in lowering plasma triglyceride levels and elevating HDL/LDL ratio. Moreover, Bihamta et al. (2017) revealed that PSO pretreatment at concentrations of 50, 100, and 200 μg/mL increased H9c2 cell viability significantly to 60% ± 2.1% (*p* < 0.01), 67% ± 2.7% (*p* < 0.001), 80.25% ± 2% (*p* < 0.001), and 88% ± 1.9% (*p* < 0.001), respectively. The findings highlight the protective effect of PSO against oxidative stress-induced cardiomyocyte damage. The protective mechanism involves the reduction of ROS production and lipid peroxidation, indicating the potential of PSO as a natural cardioprotective agent for preventing cardiovascular diseases.

### 6.6 Anti-obesity effects

PSO supplementation has also shown promising results in mitigating diet-induced obesity and insulin resistance in mice. Vroegrijk et al. (2011) conducted an *in vivo* experiment where mice were subjected to the same high-fat diet as controls, but with 1 g of fat per 100 g of food replaced by pomegranate seed oil. Over a 12-week high-fat dietary intervention, mice receiving PSO exhibited lower body weights, measuring 30.5 ± 2.9 g compared to 33.8 ± 3.2 g for controls (*p* = 0.02). This reduction in body weight was primarily attributed to a decrease in body fat mass, measuring 3.3 ± 2.3 g and 6.7 ± 2.7 g, respectively (*p* = 0.02). Notably, the insulin clamp assay revealed that PSO supplementation did not impact hepatic insulin sensitivity but significantly improved peripheral insulin sensitivity by 164% ± 52% and 92% ± 24%, respectively (*p* = 0.01). PSO has been found to decrease visceral adipose tissue weight in rodents, associated with enhanced hepatic fatty acid β-oxidation. Furthermore, it combats obesity-associated inflammation and insulin resistance by activating PPARγ receptors (Hontecillas et al., 2009). PSO also regulates body weight gain in high-fat diet-fed mice by up-regulating the expression of the beige adipose tissue-associated gene uncoupling protein 1 (UCP1). This action promotes the formation of white adipose beige-like tissue, thus controlling the size of lipid droplets in adipose tissue cells and influencing abdominal fat mass and the abdominal fat ratio (Guo et al., 2019).

Additionally, a combination of PSO and perilla seed oil has been shown to enhance blood lipids and aid in body weight management in mice (Li et al., 2015). Amri et al. (2017) conducted research on the beneficial effects of PSO with regard to brain cholinesterase activity, brain oxidative stress, and lipid profile in rats subjected to a high-fat, high-fructose diet (HFD), in both *in vivo* and *in vitro* experiments. Their findings revealed a dose-dependent inhibitory effect of PSO on cholinesterase activity. In the *in vivo* study, the HFD regimen induced a significant 17.4% increase in brain cholinesterase activity compared to normal rats. However, treatment with PSO decreased brain cholinesterase activity by 15.48% in HFD rats compared to their untreated counterparts. Furthermore, PSO regulated lipid distribution in the bloodstream and prevented lipid accumulation in both cerebral and somatic tissues relative to in untreated HFD rats. Moreover, administration of the extracts shielded the brain from oxidative stress by reducing malondialdehyde (MDA) and protein carbonylation levels while increasing SOD and glutathione peroxidase (GPx) levels. The results suggest that PSE possesses neuroprotective effects, possibly attributed to cholinesterase inhibition and antioxidant capacity enhancement.

### 6.7 Anti-microbial effects

Wound infection stands as a pivotal postoperative complication in surgical patients, with the dwindling efficacy of anti-microbial chemicals and antibiotics due to drug resistance. Consequently, the exploration of alternative natural compounds or metabolites for controlling wound infections has garnered significant attention. Recent studies have demonstrated that PSE combined with electrostatically spun poly (vinyl alcohol) (PVA) inhibits the growth of *Staphylococcus aureus* and expedites wound healing. This PVA nanofiber dressing, infused with an herbal extract possessing antibacterial and antioxidant properties, holds promise as a substitute for antibiotics in managing wound infections (Reisi et al., 2023). Treatment parameters were evaluated through *in vivo* experiments involving the application of PSO to post-excision wounds in rats once daily for 14 days. The findings indicate that PSO partially aids in treating excision wounds in rats and may be suitable for clinical treatment in humans, albeit requiring large-scale controlled studies (Atsü Md et al., 2023). Furthermore, an effective dental scaffold, crafted with PSE, pomegranate peel extract (PPE), polyvinyl alcohol, and starch using 3D printing technology, has been developed for treating periodontal disease. This scaffold covers the damaged area and contributes to healing. The incorporation of PSE and PPE enhances bacteriostatic activity against *S. aureus* and *Enterococcus faecalis*, thereby improving anti-microbial efficacy and demonstrating promising potential for clinical applications (Karabulut et al., 2023).

## 7 Discussion

**FIGURE 4:**
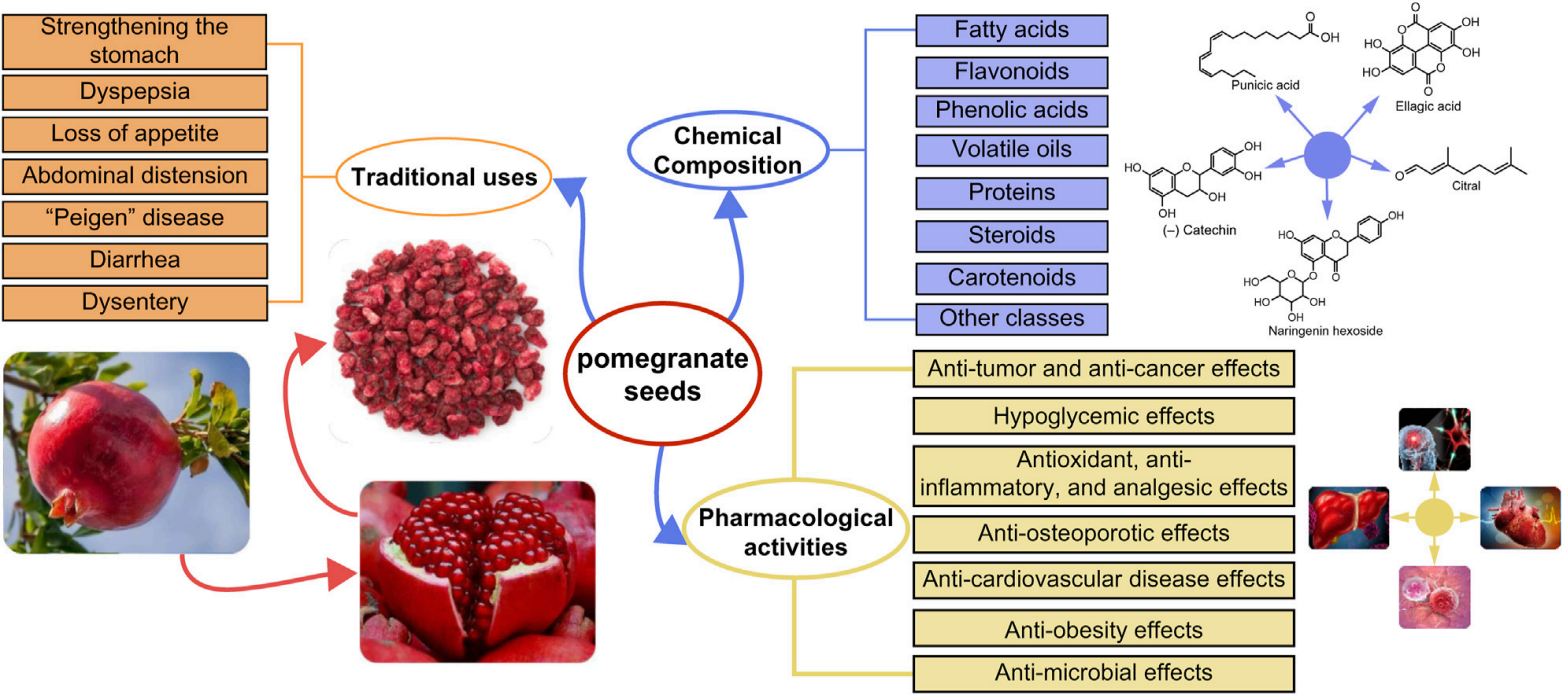
Summary of the traditional uses, chemical composition and pharmacological activities of pomegranate seeds.

This study presents a systematic review of the traditional uses and chemical composition of pomegranate seeds (PS) and the current literature on its pharmacological effects (Figure 4). PS is a by-product of pomegranate processing, and China serves as the primary producer of pomegranates. Currently, China’s utilization of PS remains relatively low, leading to significant waste generation from pomegranate cultivation and processing. PS and PSO, are abundant in fatty acids, flavonoids, sterols, tocopherols, and other active phytochemicals and metabolites. Bioactive compounds such as PA found in PSs exhibit potential to inhibit breast cancer and glioblastoma, reducing the risk of type II diabetes mellitus, and addressing insulin resistance. Understanding the chemical composition of these seeds can facilitate the exploration of their potential applications, leading to the development of valuable products and offering insights for the utilization of other plant seeds. Therefore, it is imperative to explore strategies for the development and effective utilization of the medicinal potential of PS as a valuable resource to enhance its utilization rate. Moreover, several studies have demonstrated the antioxidant, anti-diabetic, anti-obesity, anti-microbial, and anti-inflammatory properties of PS, along with its beneficial effects against cardiovascular diseases, breast cancer, prostate cancer, and osteoporosis. However, despite these findings, the potential of PS in disease prevention and treatment, as well as the molecular targets underlying its pharmacodynamic effects, remain unclear. The unknown relationship between the chemical composition and pharmacological activity of PS hampers its further development and application. To address the gap, there is a need for more in-depth exploration of the relationship between the bioactive compounds and metabolites in PS and their mechanisms of action. This requires extensive *in vivo* and *in vitro* research work, as well as clinical analyses, to assess the correlation between the chemical composition of PS and its mechanisms of action in treating various diseases. Although some experimental data support the pharmacological effects of PS, its safety and efficacy are yet to be verified adequately. Consequently, it is essential to gather sufficient safety and efficacy data to support its route of administration, dosage, and duration of administration. Ultimately, verifying the therapeutic efficacy of approved actives in animal models through human trials is crucial for validating their clinical use.

Currently, obtaining pure substances for chemical composition analysis of herbal medicines and natural products requires time-consuming and laborious sample extraction and purification processes. Identifying bioactive metabolites from traditional medicinal botanical drugs poses a challenge in developing new medicines. Notably, extracting active metabolites from PS also encounters these obstacles. Additionally, the bioavailability of some active metabolites in PS is limited. Therefore, further research is necessary to enhance both extraction techniques and the bioavailability of active metabolites. Advanced drug delivery systems such as micelles, liposomes, nanoparticles, and nanoemulsions offer promising avenues for improvement. PSO nanoemulsions have demonstrated potent anti-tumor activity by inducing apoptosis in tumor cells through DNA breaks, suggesting their potential as an alternative to chemotherapy (Mohamed et al., 2023). Sahafi et al. (2021) utilized ultrasonic emulsification to create PSO nanoemulsions loaded with alpha-tocopherol, revealing their favorable properties even in harsh environmental conditions. In a study by Petrou et al. (2021), the effects of the brain-targeted PSO nanoformulation GranaGard were investigated in patients with multiple sclerosis (MS). They concluded that GranaGard administration may enhance and stabilize cognitive dysfunction in MS patients, although further confirmation through a larger, randomized clinical trial with longer follow-up is necessary. This finding is particularly significant given the neglected research area of cognitive impairment in MS, where no approved drugs effectively address this issue. Hence, additional research is warranted to enhance both the extraction and bioavailability of active metabolites.

As modern pharmacological research advances, the scientific basis of traditional medicinal efficacy becomes increasingly clear. Traditional Chinese medicine (TCM) is gradually validated by modern science, enriching the development of contemporary medicine with TCM principles and experiences. However, some traditional applications of PS lack support from modern pharmacological research. For example, according to the Jing Zhu Ben Cao, PS is purported to treat stomach diseases, warm the stomach, and address Peigen disease. While traditional PS formulations such as “Jiebai Pills,” “Shi Wei Hei Bing Pian Wan,” and “Shiyiwei Golden Pills” are practically used for gastrointestinal disorders, contemporary pharmacological studies have predominantly explored PS’s efficacy in treating cardiovascular diseases, cancer, and osteoporosis. Notably, there is a lack of pharmacological research on the role of PS in the treatment of gastric diseases. It is suggested that modern pharmacology can be employed to explore the relevant mechanism pathways and pharmacological metabolites of PS in traditional clinically applied formulas. By doing so, we can interpret the principles of TCM with modern science while adhering to TCM’s fundamental principles. This approach facilitates the integration and compatibility of TCM with modern science, which is essential for advancing scientific research and innovation in Chinese medicine. In conclusion, PS represents a sustainable residue that merits development for its utilization. With a long history as a traditional medicine and being a natural remedy, PS holds vast potential for the prevention and treatment of various diseases. Moreover, it serves as a foundational basis for the comprehensive development of PS in the food, nutraceuticals, pharmaceuticals, and cosmetics industries.

Pomegranate is currently listed in China’s Catalog of Medicinal and Food Supplements, recognized for its dual role as both a medicinal herbal remedy and a health food supplement. Beyond its culinary use, researchers worldwide are increasingly investigating its medicinal properties and applications in the food and nutraceutical industries. Pomegranate fruit is not only edible but also utilized in beverage production (Mohagheghi et al., 2011). Pomegranate fruit extract, known for its tannins and astringent qualities, is utilized in hair color cosmetics as a metabolite. Pomegranate fruit, flowers, and rinds find common application in cosmetics (Liu et al., 2009). Pomegranate peel waste serves as feed for ruminants, while extracts from its flowers are employed for reducing injuries and swelling, and as dyes in cosmetics and textile (Jurenka, 2008). PSEs are also under consideration for wound healing in chitosan dressings (do Nascimento et al., 2020). Furthermore, PSO stands as a functional metabolite with potential applications in the fruit juice and beverage industry. While PSO is commonly utilized as a food metabolite or additive in lubricants, fuels, and paint formulations, seed oils have recently garnered increased attention due to their rich content of hydrophilic and lipophilic bioactive metabolites. These compounds hold substantial potential for nutritional, pharmaceutical, and cosmetic applications (Kalamara et al., 2015). Numerous studies have highlighted the ability of polyunsaturated fatty acids (PUFAs), particularly PA, to inhibit breast cancer and glioblastoma, as well as to reduce the risk of type II diabetes mellitus and insulin resistance. In addition to PA, PS contains a wealth of phenolic metabolites, with ellagic acid being a prominent example. This compound exhibits significant antioxidant, anti-inflammatory, and anti-microbial properties. Supplementing animal feed with ellagic acid has been shown to enhance animal performance, improve meat quality, enhance disease resistance, holding potential for application in the animal husbandry industry (Huang et al., 2024). Additionally, PSO contains phytosterols, notably β-sitosterol. Owing to its chemical composition resembling that of cholesterol, β-sitosterol finds applications across diverse fields including medicine, agriculture, and chemical industries, owing to its unique biological and physicochemical properties (Bao et al., 2022). Furthermore, PSO contains high levels of tocopherols, which are potent antioxidants that protect fats and oils from degradation and mitigate oxidative stress in the body after consumption (Barouh et al., 2022). Owing to their robust antioxidant properties, tocopherols have been suggested to reduce the risk of cancer (Das Gupta and Suh, 2016). In addition, PSO contains high levels of triterpenes, particularly squalene, a well-known dietary supplement for reducing cholesterol and triglyceride levels. Although squalene is primarily extracted from fish, it can also be found in oil-rich fruits and grains, making it crucial in vegan diets as a substitute for animal-derived sources (Caligiani et al., 2010).

**FIGURE 5:**
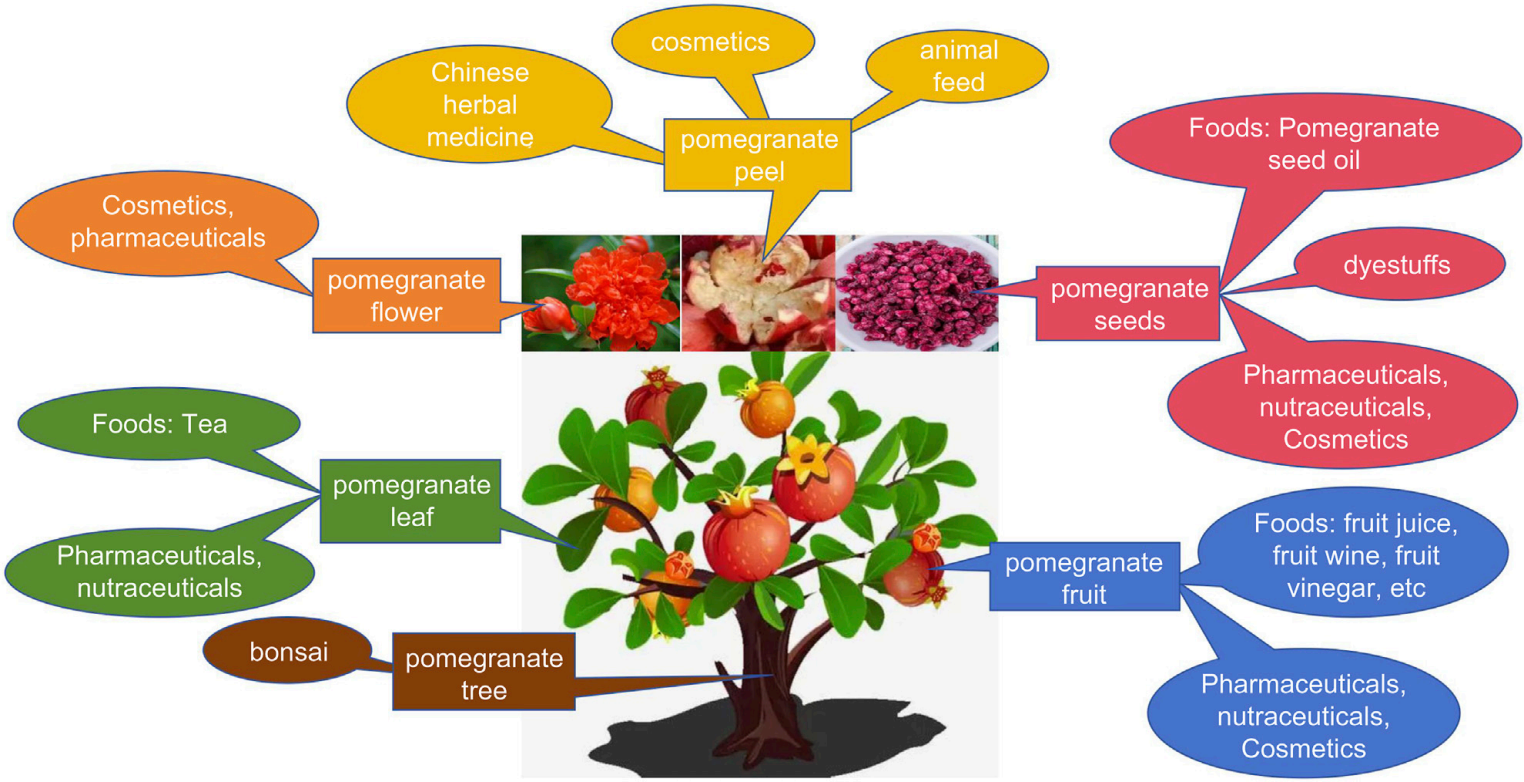
Integrated development and application of pomegranate resources.

Understanding the chemical composition of pomegranate seeds holds promise for their potential applications in developing valuable products and inspiring the utilization of other seeds. Our study outlines the comprehensive development of pomegranate resources, as illustrated in Figure 5. Ancillary industries related to pomegranates continue to emerge, substantially enhancing the economic benefits of the pomegranate industry and extending its value chain. This research serves as a model for developing the pomegranate industry chain, offering guidance and insights to enhance its competitiveness and ensure sustainable growth.
