# Supplementary material for: Pomegranate seeds: a comprehensive review of traditional uses, chemical composition, and pharmacological properties
Source: Front Pharmacol. 2024 Jul 11;15:1401826. doi: 10.3389/fphar.2024.1401826 (PMC11269133; doi:10.3389/fphar.2024.1401826)
Supplement: Supplementary file 2 [file Table1.DOCX]

Supplementary Material

**Supplementary Table 1.** Prescription and Chinese patent medicines containing pomegranate seeds

| Composition | Clinical use | Citation |
| --- | --- | --- |
| Pomegranate seed, *Cinnamomum cassia*, *Piper longum*, safflower, and round cardamom | Treatment of gastroenteritis | Kong and Luo (2021) |
| Pomegranate seed, cluster mallow fruit, *Cinnamomum cassia*, radix asparagi, Solomon's seal, *Pleurosperum hookeri* var. Thomsonii, *Piper longum*, *Mirablis himalaica*, safflower, fruit of puncture vine, and round cardamom | Warming and invigorating the stomach and kidneys; treatment of dyspepsia, cold pain in the waist and legs, frequent incontinence of urination, edema in the dorsum of the feet, impotence, and spontaneous emission | Chinese Pharmacopoeia Commission (1995) |
| Pomegranate seed, pomegranate peel, *Ziziphus montana* root bark, and purslane (fresh produce) | Treatment of pediatric enteritis | Chen (1999) |
| Myrobalan, *Aconitum tanguticum* (Maxim.) Stapf, *Aconitum pendulum* Busch, pomegranate seed, *Embelia laeta*, rose, Herpetospermum, musk, *Hypecoum erectum*, etc. | Treatment of cholecystitis | Pharmacopoeia Committee of the Ministry of Health of the People’s Republic of China (1995) |
| Pomegranate seed, *Cinnamomum cassia*, nutmeg, safflower, and *Caesalpinia crista* | Treatment of leucorrhea | Li et al. (2023b) |
| *Rhododendron anthopogonoide*, pomegranate seed, safflower, travertine, *Cinnamomum cassia*, banksia rose, agalloch eaglewood, licorice, Fructus Choerospondiatis, *Piper longum*, raisin, crab, round cardamom, clove, nutmeg, etc. | Invigorating qi and promoting digestion; treatment of diuresis and cough, headache, dizziness, sputum production, wheezing, bloating, abdominal pain, edema, acute high-altitude disease, and chronic high-altitude disease caused by long-term hypoxia | Yang (2023) |
| *Astragalus membranaceus*, dark plum, pomegranate seed, *Entada phaseoloides*, *Phyllanthus emblica*, pepper tree prickly ash, gambir plant, southern curcuma, and licorice | Strengthening the spleen and appetite, tonifying qi and consolidating the foundation, warming the middle-jiao to alleviate pain, clearing away heat and toxic materials, clearing the heart, and relieving restlessness | Chen et al. (2023b) |
| Podophyllum, pomegranate seed, *Cinnamomum cassia*, *Corydalis pygmaea*, Radix Inulae, myrobalan, bear gall, *Mirablis himalaica*, *Phyllanthus emblica*, Dalbergia wood, etc. | Clearing away heat and toxic materials; dispelling pathogenic wind and relieving pain; regulating menstrual blood; treatment of cervicitis, pelvic inflammatory disease, vaginitis, and menstrual disorders | Zhao et al. (2023) |
| *Swertia chirayita*, *Aconitum tanguticum* (Maxim.) Stapf, *Chrysosplenium*, pomegranate seed, Hei-Bing-Pian, herpetospermum, vladimiria root, *Hypecoum erectum*, Berberidis Cortex, myrobalan, Herba Lagotis, Wu ling zhi, vermiculitum, etc. | Clearing liver and gallbladder dampness heat, soothing the gallbladder and expelling stones, and reducing jaundice. Used in clinical practice for common liver and gallbladder diseases, such as liver and gallbladder heat, cholecystitis, cholelithiasis, and common bile duct stones | Chen et al. (2022) |
| Turquois, pearl, Halite Violaceous, pomegranate seed, iron powder, *Aconitum tanguticum* (Maxim.) Stapf, white sandalwood, bezoar, herpetospermum, *Cassia obtusifolia*, etc. | Soothing the liver and gallbladder, dispelling blood stasis and relieving pain, treatment of chest and rib pain caused by liver depression and qi stagnation, damp heat and stasis obstruction, vomiting and hiccups, loss of appetite, and acute and chronic hepatitis | Liu et al. (2022) |
| Pomegranate seed, cassia, *Piper longum*, round cardamom, and dried ginger | Treatment of stomach cold, bloating, indigestion, cold hands and feet, and kidney and waist pain | Pharmacopoeia Committee of the Ministry of Health of the People’s Republic of China (1995) |
| Pomegranate seed, cassia, *Piper longum*, round cardamom, and safflower | Treatment of indigestion, loss of appetite, and cold diarrhea | Pharmacopoeia Committee of the Ministry of Health of the People’s Republic of China (1995) |
| Myrobalan, pomegranate seed, *Phyllanthus emblica*, *Meconopsis*, banksia rose, papaya, Radix Inulae, round cardamom, Baxiaga, *Piper longum*, herpetospermum, Dalbergia wood, and bezoar, etc. | Regulating Qi, relieving pain, and strengthening the spleen and stomach | Huang (2022) |
| Pomegranate seed, *Cinnamomum cassia*, round cardamom, *Piper longum*, herpetospermum, Hei-Bing-Pian (charcoal), myrobalan, and rose | Strengthening the stomach, treatment of digestion and gastrointestinal infectious diseases | Cai et al. (2020) |
| Pomegranate seed, round cardamom, *Cinnamomum cassia*, *Piper longum*, dried ginger, safflower, and Amomum tsao-ko | Treatment of “Peigen” disease and “Long” disease above the liver and stomach | Pharmacopoeia Committee of the Ministry of Health of the People’s Republic of China (1995) |

**Supplementary Table 2.** Fatty acids in pomegranate seeds

| Compd. | Chemical name | Molecular weight | Formula | Citation |
| --- | --- | --- | --- | --- |
| 1 | Punicic acid | 278.4 | C_18_H_30_O_2_ | Zielińska et al. (2022); Iriti et al. (2023); Eikani et al. (2012); Siano et al. (2016); Verardo et al. (2014); Harzallah et al. (2016); Loukhmas et al. (2021); Yang et al. (2013); Fu et al. (2015); Peng (2019) |
| 2 | α-Eleostearic acid | 278.4 | C_18_H_30_O_2_ | Siano et al. (2016); Harzallah et al. (2016) |
| 3 | cis-α-Linolenic acid | 278.4 | C_18_H_30_O_2_ | Siano et al. (2016); Harzallah et al. (2016); Loukhmas et al. (2021); Fu et al. (2015); Peng (2019) |
| 4 | cis-γ-Linolenic acid | 278.4 | C_18_H_30_O_2_ | Harzallah et al. (2016); Fu et al. (2015) |
| 5 | Calendic acid | 278.4 | C_18_H_30_O_2_ | Harzallah et al. (2016) |
| 6 | trans-9, trans-11, cis-13-Octadecatrienoic acid | 278.4 | C_18_H_30_O_2_ | Siano et al. (2016); Harzallah et al. (2016) |
| 7 | Catalpic acid | 278.4 | C_18_H_30_O_2_ | Siano et al. (2016) |
| 8 | cis-9-Palmitoleic acid | 254.41 | C_16_H_30_O_2_ | Eikani et al. (2012); Harzallah et al. (2016); Fu et al. (2015); Wu et al. (2015a) |
| 9 | cis-Oleic acid | 282.5 | C_18_H_34_O_2_ | Zielińska et al. (2022); Iriti et al. (2023); Eikani et al. (2012); Verardo et al. (2014); Harzallah et al. (2016); Loukhmas et al. (2021); Yang et al. (2013); Fu et al. (2015), Wu et al. (2015a); Du et al. (2017); Fathy et al. (2021) |
| 10 | trans-Elaidic acid | 282.5 | C_18_H_34_O_2_ | Iriti et al. (2023); Harzallah et al. (2016) |
| 11 | cis-cis-Linoleic acid | 280.4 | C_18_H_32_O_2_ | Iriti et al. (2023); Eikani et al. (2012); Siano et al. (2016); Harzallah et al. (2016); Du et al. (2017) |
| 12 | trans-9, cis-12-Octadecadienoic acid | 280.4 | C_18_H_32_O_2_ | Kaseke et al. (2021) |
| 13 | trans-10, cis-12-Octadecadienoic acid | 280.4 | C_18_H_32_O_2_ | Kaseke et al. (2021) |
| 14 | Gadoleic acid | 310.5 | C_20_H_38_O_2_ | Iriti et al. (2023); Peng (2019); Wu et al. (2015a); Kaseke et al. (2021) |
| 15 | cis-9,trans-12-Octadecadienoic acid | 280.4 | C_18_H_32_O_2_ | Kaseke et al. (2021) |
| 16 | cis-Myristoleic acid | 226.35 | C_14_H_26_O_2_ | Harzallah et al. (2016) |
| 17 | trans-9-Palmitoleic acid | 254.41 | C_16_H_30_O_2_ | Harzallah et al. (2016); Fu et al. (2015) |
| 18 | Eicosatrienoic acid | 360.5 | C_20_H_34_O_2_ | Harzallah et al. (2016) |
| 19 | Methyl (E)-11-eicosenoate | 324.5 | C_21_H_40_O_2_ | Li et al. (2023a); Đurđević et al. (2018) |
| 20 | Nervonic acid | 366.6 | C_24_H_46_O_2_ | Kaseke et al. (2021) |
| 21 | Methyl (Z)-11-eicosenoate | 324 | C_21_H_40_O_2_ | Đurđević et al. (2018) |
| 22 | Methyl linolelaidate | 294 | C_19_H_34_O_2_ | Đurđević et al. (2018) |
| 23 | Methyl elaidate | 296 | C_19_H_36_O_2_ | Đurđević et al. (2018) |
| 24 | Methyl linoleate | 294 | C_19_H_34_O_2_ | Đurđević et al. (2018) |
| 25 | Methyl punicate | 292 | C_19_H_32_O_2_ | Đurđević et al. (2018) |
| 26 | Lecithin | 766.1 | C_44_H_80_NO_7_P | Eikani et al. (2012) |
| 27 | trans-Elaidic acid | 282.5 | C_18_H_34_O_2_ | Iriti et al. (2023); Harzallah et al. (2016) |
| 28 | Eicosadienoic acid | 308.5 | C_20_H_36_O_2_ | Kaseke et al. (2021) |
| 29 | Phosphatidylethanolamine | 299.2 | C_9_H_18_NO_8_P | Eikani et al. (2012) |
| 30 | Phosphatidylcholine | 257.2 | C_8_H_2_0NO_6_P | Eikani et al. (2012) |
| 31 | Lysophosphatidylethanolamine | 256.17 | C_7_H_15_NO_7_P^-^ | Eikani et al. (2012) |
| 32 | Lauric acid | 200.32 | C_12_H_24_O_2_ | Harzallah et al. (2016) |
| 33 | Myristic acid | 228.37 | C_14_H_28_O_2_ | Li et al. (2023a); Verardo et al. (2014); Harzallah et al. (2016); Fu et al. (2015); Wu et al. (2015a); |
| 34 | Palmitic acid | 256.42 | C_16_H_32_O_2_ | Li et al. (2023a); Iriti et al. (2023); Eikani et al. (2012); Siano et al. (2016); Verardo et al. (2014), Harzallah et al. (2016); Loukhmas et al. (2021); Yang et al. (2013); Fu et al. (2015); Wu et al. (2015a); Du et al. (2017) |
| 35 | Margaric acid | 270.5 | C_17_H_34_O_2_ | Iriti et al. (2023); Siano et al. (2016); Verardo et al. (2014); Peng (2019); Wu et al. (2015a); Du et al. (2017) |
| 36 | Stearic acid | 284.5 | C_18_H_36_O_2_ | Zielińska et al. (2022); Li et al. (2023a); Iriti et al. (2023); Eikani et al. (2012); Siano et al. (2016); Verardo et al. (2014); Harzallah et al. (2016); Yang et al. (2013); Fu et al. (2015); Peng (2019); Wu et al. (2015a); Du et al. (2017) |
| 37 | Arachidic acid | 312.5 | C_20_H_40_O_2_ | Li et al. (2023a); Iriti et al. (2023); Eikani et al. (2012); Siano et al. (2016); Verardo et al. (2014); Harzallah et al. (2016); Fu et al. (2015); Peng (2019); Wu et al. (2015a); Du et al. (2017) |
| 38 | Behenic acid | 340.6 | [C](https://pubchem.ncbi.nlm.nih.gov/)_[22](https://pubchem.ncbi.nlm.nih.gov/)_[H](https://pubchem.ncbi.nlm.nih.gov/)_[44](https://pubchem.ncbi.nlm.nih.gov/)_[O](https://pubchem.ncbi.nlm.nih.gov/)_[2](https://pubchem.ncbi.nlm.nih.gov/)_ | Li et al. (2023a); Iriti et al. (2023); Verardo et al. (2014); Harzallah et al. (2016); Peng (2019); Wu et al. (2015a) |
| 39 | Lignoceric acid | 368.6 | C_24_H_48_O_2_ | Li et al. (2023a); Siano et al. (2016); Harzallah et al. (2016); Fu et al. (2015); Wu et al. (2015a) |
| 40 | Methyl palmitate | 270 .5 | C_17_H_34_O_2_ | Đurđević et al. (2018) |
| 41 | Methyl margarate | 284 | C_18_H_36_O_2_ | Đurđević et al. (2018) |
| 42 | Methyl stearate | 298.5 | C_19_H_38_O_2_ | Peng (2019); Đurđević et al. (2018) |
| 43 | Methyl arachidate | 326.5 | C_21_H_42_O_2_ | Đurđević et al. (2018) |
| 44 | Methyl behenate | 354 .6 | C_23_H_46_O_2_ | Đurđević et al. (2018) |
| 45 | Methyl lignocerate | 382.6 | C_25_H_50_O_2_ | Đurđević et al. (2018) |

**Supplementary Table 3.** Flavonoids in pomegranate seeds

| Compd. | Chemical name | Molecular weight | Formula | Citation |
| --- | --- | --- | --- | --- |
| 46 | 2′,5′-Dimethoxyflavone | 282.29 | C_17_H_14_O_4_ | Fathy et al. (2021) |
| 47 | 7,2′,3′-Trimethoxyflavone | 312.32 | C_18_H_16_O_5_ | Fathy et al. (2021) |
| 48 | 3,2′,4′,5′,6-Pentamethoxyflavone | 372.37 | C_20_H_2_0O_7_ | Fathy et al. (2021) |
| 49 | 3,6,2′,3′-Tetramethoxyflavone | 342.34 | C_19_H_18_O_6_ | Fathy et al. (2021) |
| 50 | 2′,3′-Dimethoxyflavone | 282.29 | C_17_H_14_O_4_ | Fathy et al. (2021) |
| 51 | 6,7,3′,4′-Tetramethoxyflavone | 342.35 | C_19_H_18_O_6_ | Fathy et al. (2021) |
| 52 | 3,5,7,3′,4′-Pentamethoxyflavone | 372.37 | C_20_H_2_0O_7_ | Fathy et al. (2021) |
| 53 | 3,2′,4′,5′-Tetramethoxyflavone | 342.35 | C_19_H_18_O_6_ | Fathy et al. (2021) |
| 54 | 5-Hydroxy-3′,4′,5′,6,7,8-hexamethoxyflavone | 418.39 | C_21_H_22_O_9_ | Fathy et al. (2021) |
| 55 | 7,3′,4′,5′-Tetramethoxyflavone | 342.35 | C_19_H_18_O_6_ | Fathy et al. (2021) |
| 56 | 3,7,8,2′-Tetramethoxyflavone | 342.35 | C_19_H_18_O_6_ | Fathy et al. (2021) |
| 57 | 3,6,3′,4′-Tetramethoxyflavone | 342.35 | C_19_H_18_O_6_ | Fathy et al. (2021) |
| 58 | 3,5,7-Trimethoxyflavone | 312.32 | C_18_H_16_O_5_ | Fathy et al. (2021) |
| 59 | 3-Hydroxy-7,8,2′-trimethoxyflavone | 328.32 | C_18_H_16_O_6_ | Fathy et al. (2021) |
| 60 | 7,8,3′,4′-Tetramethoxyflavone | 342.35 | C_19_H_18_O_6_ | Fathy et al. (2021) |
| 61 | Kaempferol | 286.24 | C_15_H_10_O_6_ | Li et al. (2020); Khemakhem et al. (2021) |
| 62 | Apigenin | 270.24 | C_15_H_10_O_5_ | Li et al. (2020); Khemakhem et al. (2021) |
| 63 | Quercetin | 302.24 | C_15_H_10_O_7_ | Li et al. (2020); Khemakhem et al. (2021) |
| 64 | Luteolin | 286.24 | C_15_H_10_O_6_ | Li et al. (2020); Khemakhem et al. (2021) |
| 65 | Nobiletin | 402.40 | C_21_H_22_O_8_ | Fathy et al. (2021) |
| 66 | Scutellarein | 286.24 | C_15_H_14_O_6_ | Li et al. (2020) |
| 67 | Myricetin | 318.24 | C_15_H_10_O_8_ | Li et al. (2020) |
| 68 | (+)-Catechin | 290.27 | C_15_H_14_O_6_ | Ambigaipalan et al. (2017); Khemakhem et al. (2021) |
| 69 | Cianidanol | 290.27 | C_15_H_14_O_6_ | Li et al. (2020) |
| 70 | Gallocatechin | 306.27 | C_15_H_14_O_7_ | Li et al. (2020) |
| 71 | Epigallocatechin | 306.27 | C_15_H_14_O_7_ | Li et al. (2020) |
| 72 | Genistein | 270.24 | C_15_H_10_O_5_ | Li et al. (2020) |
| 73 | Kaempferol 3-  O-rutinoside | 594.52 | C_27_H_30_O_15_ | Khemakhem et al. (2021) |
| 74 | Rutin | 610.52 | C_27_H_30_O_16_ | Li et al. (2020); Khemakhem et al. (2021) |
| 75 | Kaempferol 3-O-glucoside | 484.38 | C_21_H_2_0O_11_ | Ambigaipalan et al. (2017) |
| 76 | Isoquercitrin | 464.4 | C_21_H_2_0O_12_ | Ambigaipalan et al. (2017); Khemakhem et al. (2021) |
| 77 | Lonicerin | 594.52 | C_27_H_30_O_15_ | Li et al. (2020) |
| 78 | Epicatechin-3-O-gallat | 442.38 | C_22_H_18_O_10_ | Khemakhem et al. (2021) |
| 79 | Quercetin hexoside | 464.38 | C_21_H_2_0O_12_ | Ambigaipalan et al. (2017) |
| 80 | Cosmosiin | 432.38 | C_21_H_2_0O_10_ | Li et al. (2020) |
| 81 | Kaempferol-7-O-glucoside | 448.38 | C_21_H_2_0O_11_ | Li et al. (2020) |
| 82 | Cynaroside | 448.38 | C_21_H_2_0O_11_ | Li et al. (2020) |
| 83 | Genistin | 432.38 | C_21_H_2_0O_10_ | Li et al. (2020) |
| 84 | Oroxin A | 432.38 | C_21_H_2_0O_10_ | Li et al. (2020) |
| 85 | Baicalin | 446.36 | C_21_H_18_O_11_ | Li et al. (2020) |
| 86 | Hyperoside | 464.38 | C_21_H_2_0O_12_ | Li et al. (2020) |
| 87 | Trans-dihydrokaempferol hexoside | 450.40 | C_21_H_22_O_11_ | Ambigaipalan et al. (2017) |
| 88 | Kaempferol-3-O-glucorhamnoside | 594.57 | C_27_H_30_O_15_ | Li et al. (2020) |
| 89 | Liquiritin | 418.40 | C_21_H_22_O_9_ | Li et al. (2020) |
| 90 | Naringenin hexoside | 434.4 | C_21_H_22_O_10_ | Ambigaipalan et al. (2017) |
| 91 | Pinocembrin | 256.26 | C_15_H_12_O_4_ | Li et al. (2020) |
| 92 | Naringetol | 272.26 | C_15_H_12_O_5_ | Li et al. (2020) |
| 93 | Eriodictyol | 288.25 | C_15_H_12_O_6_ | Li et al. (2020) |
| 94 | Dihydromyricetin | 320.25 | C_15_H_12_O_8_ | Li et al. (2020) |
| 95 | Dihydrokaempferol | 288.25 | C_15_H_12_O_6_ | Hernández-Corroto et al. (2022) |
| 96 | Naringenin chalcone | 272.26 | C_15_H_12_O_5_ | Li et al. (2020) |
| 97 | Phloretin | 274.27 | C_15_H_14_O_5_ | Li et al. (2020) |

**Supplementary Table 4.** Phenolic metabolites other than flavonoids in pomegranate seeds

| Compd. | Chemical name | Molecular weight | Formula | Citation |
| --- | --- | --- | --- | --- |
| 98 | Gallic acid | 170.12 | C_7_H_6_O_5_ | Li et al. (2020); Hernández-Corroto et al. (2022); Fathy et al. (2021); Khemakhem et al. (2021) |
| 99 | Vanillic acid | 168.15 | C_8_H_8_O_4_ | Hernández-Corroto et al. (2022); Ambigaipalan et al. (2017) |
| 100 | Methyl gallate | 184.15 | C_8_H_8_O_5_ | Li et al. (2020); Hernández-Corroto et al. (2022) |
| 101 | Ethyl gallate | 198.17 | C_9_H_10_O_5_ | Hernández-Corroto et al. (2022); |
| 102 | Protocatechuic acid | 154.12 | C_7_H_6_O_4_ | Li et al. (2020); Ambigaipalan et al. (2017); Khemakhem et al. (2021) |
| 103 | 2,5-Dihydroxybenzoic acid | 154.12 | C_7_H_6_O_4_ | Li et al. (2020) |
| 104 | Propyl gallate | 212.20 | C_10_H_12_O_5_ | Fathy et al. (2021) |
| 105 | Paeonol | 166.18 | C_9_H_10_O_3_ | Li et al. (2020) |
| 106 | Cinnamic acid | 148.16 | C_9_H_8_O_2_ | Li et al. (2020) |
| 107 | p-Coumaric acid | 164.16 | C_9_H_8_O_3_ | Ambigaipalan et al. (2017); Khemakhem et al. (2021) |
| 108 | Caffeic acid | 180.16 | C_9_H_8_O_4_ | Li et al. (2020); Hernández-Corroto et al. (2022) |
| 109 | Ferulic acid | 194.18 | C_10_H_10_O_4_ | Li et al. (2020); Ambigaipalan et al. (2017); Khemakhem et al. (2021) |
| 110 | Isoferulic acid | 194.19 | C_10_H_10_O_4_ | Li et al. (2020) |
| 111 | Methyl ferulate | 208.21 | C_11_H_12_O_4_ | Li et al. (2020) |
| 112 | Sinapic acid | 224.21 | C_11_H_12_O_5_ | Li et al. (2020); Khemakhem et al. (2021) |
| 113 | Ellagic acid | 302.19 | C_14_H_6_O_8_ | Li et al. (2020); Hernández-Corroto et al. (2022); Fathy et al. (2021); Ambigaipalan et al. (2017) |
| 114 | Ellagic acid pentoside | 434.31 | C_19_H_14_O_12_ | Hernández-Corroto et al. (2022); Ambigaipalan et al. (2017) |
| 115 | Valoneic acid dilactone | 470.30 | C_21_H_10_O_13_ | Hernández-Corroto et al. (2022) |
| 116 | Ellagic acid deoxyhexoside | 448.34 | C_20_H_16_O_12_ | Hernández-Corroto et al. (2022) |
| 117 | Chlorogenic Acid | 354.31 | C_16_H_18_O_9_ | Khemakhem et al. (2021) |
| 118 | Ellagic acid hexoside | 464.33 | C_20_H_16_O_13_ | Hernández-Corroto et al. (2022); Ambigaipalan et al. (2017) |
| 119 | Brevifolin carboxylic acid | 292.20 | C_13_H_8_O_8_ | Li et al. (2020); Hernández-Corroto et al. (2022); Ambigaipalan et al. (2017) |
| 120 | Scoparone | 206.20 | C_11_H_10_O_4_ | Li et al. (2020) |
| 121 | Umbelliferone | 162.14 | C_9_H_6_O_3_ | Li et al. (2020) |
| 122 | 4-Methylumbelliferone | 176.17 | C_10_H_8_O_3_ | Li et al. (2020) |
| 123 | 7-Methoxycoumarin | 176.17 | C_10_H_8_O_3_ | Li et al. (2020) |
| 124 | Caffeic acid phenethyl ester | 284.31 | C_17_H_16_O_4_ | Li et al. (2020) |
| 125 | 3,4-Dihydroxybenzaldehyde | 138.12 | C_7_H_6_O_3_ | Li et al. (2020) |
| 126 | Pyrogallol | 126.11 | C_6_H_6_O_3_ | Li et al. (2020) |
| 127 | Oleuropein | 540.52 | C_25_H_32_O_13_ | Li et al. (2020) |
| 128 | Emodin-8- glucoside | 432.38 | C_21_H_2_0O_10_ | Li et al. (2020) |
| 129 | Corilagin | 634.45 | C_27_H_22_O_18_ | Li et al. (2020) |
| 130 | Pedunculagin | 784.54 | C_34_H_24_O_22_ | Hernández-Corroto et al. (2022) |
| 131 | Terflavin B | 784.54 | C_34_H_24_O_22_ | Hernández-Corroto et al. (2022) |
| 132 | Pedunculagin II | 786.56 | C_34_H_26_O_22_ | Hernández-Corroto et al. (2022) |
| 133 | Punicalagin | 1116.72 | C_48_H_28_O_32_ | Hernández-Corroto et al. (2022) |
| 134 | Granatin B | 952.65 | C_41_H_28_O_27_ | Hernández-Corroto et al. (2022) |
| 135 | Punicalin | 782.53 | C_34_H_22_O_22_ | Li et al. (2020); Hernández-Corroto et al. (2022) |

**References**

Ambigaipalan, P., De Camargo, A. C., Shahidi, F. (2017). Identification of phenolic antioxidants and bioactives of pomegranate seeds following juice extraction using HPLC-DAD-ESI-MSn. Food Chem. 221, 1883–1894. doi: [10.1016/j.foodchem.2016.10.058](https://doi.org/10.1016/j.foodchem.2016.10.058).

Cai, R. C., Pan, X., Li, K., Cairang, S. Z., Gao, X. Y., Zhang, Q. (2020). On content determination of ellagic acid and piperine in Tibetan medicine pomegranate lotus powder. Chin. Pharm. Aff. 34, 215–221.

Chen, W., Ma, T. F., Wu, H. C., Liu, Y. Y., Renqing, D. Z., Zhang, J. K., et al. (2022). Action mechanism of Tibetan medicine Shiwuwei Saierdou pill in treatment of guinea pig cholecystitis. Acta Chin. Med. 37, 1676–1687.

Chen, X. H. (1999). External treatment for infantile bloating and diarrhea. Med. Health Care. 2, 21.

Chen, Y. W., Tian, Y. Z., Lin, L., Wei, Y. T., Zhang, Q. (2023b). Effect of Sanzi prescription on postoperative efficacy, immune function and quality of life of patients treated by endoscopy for colorectal adenoma. Liaoning J. Trad. Chin. Med. 50, 119–122.

Chinese Pharmacopoeia Commission. (1995). Drug standards of the Ministry of Health of the People’s Republic of China·Tibetan medicines: Book I. Standards Press of China.

Du, J., Cao, Y., Dai, W. J., Yang, G. H. (2017). Extraction of pomegranate seed oil by low-temperature continuous phase transition and the analysis of its fatty acid. Food Mach. 33, 148–151.

Đurđević, S., Šavikin, K., Živković, J., Böhm, V., Stanojković, T., Damjanović, A., et al. (2018). Antioxidant and cytotoxic activity of fatty oil isolated by supercritical fluid extraction from microwave pretreated seeds of wild growing *Punica granatum* L. J. Supercrit. Fluids. 133, 225–232. doi: [10.1016/j.supflu.2017.10.021](https://doi.org/10.1016/j.supflu.2017.10.021).

Eikani, M. H., Golmohammad, F., Homami, S. S. (2012). Extraction of pomegranate (*Punica granatum* L.) seed oil using superheated hexane. Food Bioprod. Process. 90, 32–36. doi: [10.1016/j.fbp.2011.01.002](https://doi.org/10.1016/j.fbp.2011.01.002).

Fathy, S. M., El-Dash, H. A., Said, N. I. (2021). Neuroprotective effects of pomegranate (*Punica granatum* L.) juice and seed extract in paraquat-induced mouse model of Parkinson’s disease. BMC Complement. Med. Ther. 21, 130. doi: [10.1186/s12906-021-03298-y](https://doi.org/10.1186/s12906-021-03298-y).

Fu, G. Q., Liu, L., Zhang, L., Gao, Y., Xv, X.N., Xie, F., et al. (2015). *Punica granatum* seed oil inhibits malignant behavior of breast cancer cells. Mil. Med. Sci. 39, 438–442.

Harzallah, A., Hammami, M., Kępczyńska, M. A., Hislop, D. C., Arch, J. R. S., Cawthorne, M. A., et al. (2016). Comparison of potential preventive effects of pomegranate flower, peel and seed oil on insulin resistance and inflammation in high-fat and high-sucrose diet-induced obesity mice model. Arch. Physiol. Biochem. 122, 75–87. doi: [10.3109/13813455.2016.1148053](https://doi.org/10.3109/13813455.2016.1148053).

Hernández-Corroto, E., Boussetta, N., Marina, M. L., García, M. C., Vorobiev, E. (2022). High voltage electrical discharges followed by deep eutectic solvents extraction for the valorization of pomegranate seeds (*Punica granatum* L.). Innov. Food Sci. Emerg. Technol. 79, 103055. doi: [10.1016/j.ifset.2022.103055](https://doi.org/10.1016/j.ifset.2022.103055).

Huang, Y. (2022). Clinical efficacy of cold water stone twenty-one flavour powder of Mongolian medicine in the treatment of gastric ulcer. J. Med. Pharm. Chin. Minorities. 28, 20–22.

Iriti, G., Bonacci, S., Lopreiato, V., Frisina, M., Oliverio, M., Procopio, A. (2023). Functional compounds of cold-pressed pomegranate seed oil: Fatty acids and phytosterols profile as quality biomarkers for origin discrimination. Foods. 12, 2599. doi: [10.3390/foods12132599](https://doi.org/10.3390/foods12132599).

Kaseke, T., Opara, U. L., Fawole, O. A. (2021). Quality and antioxidant properties of cold-pressed oil from blanched and microwave-pretreated pomegranate seed. Foods. 10, 712. doi: [10.3390/foods10040712](https://doi.org/10.3390/foods10040712).

Khemakhem, M., Zarroug, Y., Jabou, K., Selmi, S., Bouzouita, N. (2021). Physicochemical characterization of oil, antioxidant potential, and phenolic profile of seeds isolated from Tunisian pomegranate (Punica granatum L.) cultivars. J. Food Sci. 86, 852–859. doi: [10.1111/1750-3841.15636](https://doi.org/10.1111/1750-3841.15636).

Kong, X. W., Luo, M. Jv Y.K. Xv, J.H., Zheng, Z., Xiong, H., et al. (2021). Exploring the mechanism of action of pomegranate stomach-enhancing pills in the treatment of gastroenteritis based on network pharmacology. Modernization of Traditional Chinese Medicine and Materia Medica-World Science and Technology. 23, 1415–1427.

Li, G., Chen, J., Yang, Q., Yang, X., Wang, P., Lei, H., et al. (2023a). Identification of chemical constituents in pomegranate seeds based on ultra-high-performance supercritical fluid chromatography coupled with quadrupole time-of-flight mass spectrometry. Rapid Commun. Mass Spectrom. 37 Supplement 1, e9482. doi: [10.1002/rcm.9482](https://doi.org/10.1002/rcm.9482).

Li, G., Chen, M., Chen, J., Shang, Y., Lian, X., Wang, P., et al. (2020). Chemical composition analysis of pomegranate seeds based on ultra-high-performance liquid chromatography coupled with quadrupole-Orbitrap high-resolution mass spectrometry. J. Pharm. Biomed. Anal. 187, 113357. doi: [10.1016/j.jpba.2020.113357](https://doi.org/10.1016/j.jpba.2020.113357).

Li, Y., Zhou, D. L., Cairang, L., Zhang, M. T. (2023b). Quality standard study on Zang Medicine Liuwei Shiliu pill. Chin. J. Ethnomed. Ethnopharmacy. 32, 19–24.

Liu, X. A., Pan, L., Yang, C. H., Li, S. L., Yv, L. Q., Pu, L. L., et al. (2022). Understanding and treatment of hepatobiliary diseases in Tibetan medicine. Pharmacol. Clin. Chin. Mater. Med. 13.

Loukhmas, S., Kerak, E., Elgadi, S., Ettalibi, F., El Antari, A., Harrak, H. (2021). Oil content, fatty acid composition, physicochemical properties, and antioxidant activity of seed oils of ten Moroccan pomegranate cultivars. J. Food Qual. 2021, 1–13. doi: [10.1155/2021/6617863](https://doi.org/10.1155/2021/6617863).

Peng, Y. (2019). Comparative analysis of the biological components of pomegranate seed from different cultivars. Int. J. Food Prop. 22, 784–794. doi: [10.1080/10942912.2019.1609028](https://doi.org/10.1080/10942912.2019.1609028).

Pharmacopoeia Committee of the Ministry of Health of the People’s Republic of China. (1995). Drug Standards of the Ministry of Health of the People’s Republic of China·Tibetan Medicines: Book I (People’s Medical Publishing House).

Siano, F., Straccia, M. C., Paolucci, M., Fasulo, G., Boscaino, F., Volpe, M. G. (2016). Physico-chemical properties and fatty acid composition of pomegranate, cherry and pumpkin seed oils. J. Sci. Food Agric. 96, 1730–1735. doi: [10.1002/jsfa.7279](https://doi.org/10.1002/jsfa.7279).

Verardo, V., Garcia-Salas, P., Baldi, E., Segura-Carretero, A., Fernandez-Gutierrez, A., Caboni, M. F. (2014). Pomegranate seeds as a source of nutraceutical oil naturally rich in bioactive lipids. Food Res. Int. 65, 445–452. doi: [10.1016/j.foodres.2014.04.044](https://doi.org/10.1016/j.foodres.2014.04.044).

Wu, P. K., Lin, H. Y., Huang, J. M., Wang, C. M. (2015a). Analysis of fatty acid in the tomato, Hami melon, grape and pomegranate seed oils in Xinjiang using gas chromatography-mass spectrometry. Spec. Wild Econ. Anim. Plant Res. 37, 50–57.

Yang, W. Z., Zhou, Y., Yang, X. X., Zhou, H. F., Tan, F. X. (2013). A study on the extraction of pomegranate seed oil and its fatty acid composition. J. Inner Mongolia Univ. Technol. 32, 96–100.

Yang, X. L. (2023). Study on the Preparation and Quality Standard of the Sixteen Flavors Azalea Concentrated Pill. Anhui University of Chinese Medicine.

Zhao, X. N., Zhu, Y. R., Yuan, Z. B., Zhang, J. W., Xie, H. B. (2023). Study on antibacterial effects and mechanism of Tibetan medicine Ershiwuwei Guijiu Pills on *Staphylococcus aureus*. Drug Eval. Res. 46, 78–84.

Zielińska, A., Wójcicki, K., Klensporf-Pawlik, D., Marzec, M., Lucarini, M., Durazzo, A., et al. (2022). Cold-pressed pomegranate seed oil: Study of punicic acid properties by coupling of GC/FID and FTIR. Molecules. 27, 5863. doi: [10.3390/molecules27185863](https://doi.org/10.3390/molecules27185863).
